# Population-Specific Haplotype Association of the Postsynaptic Density Gene *DLG4* with Schizophrenia, in Family-Based Association Studies

**DOI:** 10.1371/journal.pone.0070302

**Published:** 2013-07-25

**Authors:** Shabeesh Balan, Kazuo Yamada, Eiji Hattori, Yoshimi Iwayama, Tomoko Toyota, Tetsuo Ohnishi, Motoko Maekawa, Manabu Toyoshima, Yasuhide Iwata, Katsuaki Suzuki, Mitsuru Kikuchi, Takeo Yoshikawa

**Affiliations:** 1 Laboratory for Molecular Psychiatry, RIKEN Brain Science Institute, Saitama, Japan; 2 Department of Psychiatry and Neurology, Hamamatsu University School of Medicine, Shizuoka, Japan; 3 Department of Psychiatry and Neurobiology, Kanazawa University Graduate School of Medicine, Kanazawa, Japan; Chiba University Center for Forensic Mental Health, Japan

## Abstract

The post-synaptic density (PSD) of glutamatergic synapses harbors a multitude of proteins critical for maintaining synaptic dynamics. Alteration of protein expression levels in this matrix is a marked phenomenon of neuropsychiatric disorders including schizophrenia, where cognitive functions are impaired. To investigate the genetic relationship of genes expressed in the PSD with schizophrenia, a family-based association analysis of genetic variants in PSD genes such as *DLG4*, *DLG1*, *PICK1* and *MDM2,* was performed, using Japanese samples (124 pedigrees, n = 376 subjects). Results showed a significant association of the rs17203281 variant from the *DLG4* gene, with preferential transmission of the C allele (p = 0.02), although significance disappeared after correction for multiple testing. Replication analysis of this variant, found no association in a Chinese schizophrenia cohort (293 pedigrees, n = 1163 subjects) or in a Japanese case-control sample (n = 4182 subjects). The *DLG4* expression levels between postmortem brain samples from schizophrenia patients showed no significant changes from controls. Interestingly, a five marker haplotype in *DLG4,* involving rs2242449, rs17203281, rs390200, rs222853 and rs222837, was enriched in a population specific manner, where the sequences A-C-C-C-A and G-C-C-C-A accumulated in Japanese (p = 0.0009) and Chinese (p = 0.0007) schizophrenia pedigree samples, respectively. However, this could not be replicated in case-control samples. None of the variants in other examined candidate genes showed any significant association in these samples. The current study highlights a putative role for *DLG4* in schizophrenia pathogenesis, evidenced by haplotype association, and warrants further dense screening for variants within these haplotypes.

## Introduction

Schizophrenia is a serious psychiatric disorder, with high heritability and a worldwide lifetime risk of approximately one percent [1]. Several hypotheses for disease pathogenesis have been put forward, which include abnormal functional integration of neural systems, resulting in impaired synaptic transmission and plasticity [2]–[6]. In particular, dysfunction in the glutamergic system, through the glutamate receptor which mediates excitatory neurotransmission in the central nervous system, has been implicated in the development of schizophrenia. Other relevant receptors include the ionotropic α-amino-3-hydroxy-5-ethylisoxazole-4-propionic acid receptor (AMPA), *N*-methyl-d-aspartate (NMDA) and metabotropic glutamate receptors (mGluRs). The role of glutamergic receptors in schizophrenia pathogenesis is strengthened by the findings that administering NMDA receptor antagonists can induce psychotic symptoms in human and also the aberrant receptor gene expression patterns observed in schizophrenia brain samples [7]–[9].

The dynamics of glutamate receptor trafficking to the postsynaptic membrane is affected by a series of scaffold proteins present in the protein-dense excitatory post synapses, known as the postsynaptic density (PSD). The PSD comprises cell-adhesion proteins, cytoskeletal proteins, scaffolding and adaptor proteins, membrane-bound receptors and channels, G-proteins and modulators and signaling molecules, such as kinases and phosphatases [10]. The well characterized scaffolding proteins within the PSD include PDZ [PSD95 (post-synaptic density protein 95)/DLG/ZO1] domain containing members of the PSD95 family, members of the AKAP (A-kinase anchoring protein) family, Homer family, SAPAP (SAP90/PSD95-associated protein) family and Shank (SH3 and multiple ankyrin repeat domain) family [11].

The subfamily of MAGUKs (membrane-associated guanylate kinase), comprising synapse-associated protein (SAP) 102, SAP97, PSD93 and PSD95 are of special interest with respect to their role in receptor clustering and signaling in glutamergic synapses, and also for the aberrant expression patterns observed in neuropsychiatric disorders [12]. The role of PSD proteins is to maintain synaptic plasticity, and impaired cognitive function seems to stem from altered glutamate-dependent synaptic transmission, due to compromised expression of PSD proteins [13]. This notion is further supported by reported aberrant expression levels of *DLG4* encoding PSD95, *DLG1* encoding SAP97 and *PICK1* encoding PICK1 (Protein interacting with C-kinase-1), in post mortem brain samples from schizophrenia patients, indicating defective glutamate receptor targeting and downstream signaling in schizophrenia [14], [15].

The PSD95 is primarily involved in tethering NMDA and AMPA receptors, through stargazin, to signaling proteins and the neuronal cytoskeleton of the postsynaptic membrane [16]. These proteins are also involved in channel gating, by AMPA receptor incorporation, NMDA receptor trafficking, maturation of excitatory synapses, regulation of synaptic strength and signaling [17]–[21]. Furthermore, SAP97 also interacts with NMDA and AMPA receptors, and plays a major role in clathrin-mediated endocytosis of AMPA receptors, through interaction with the GluR1 subunit [22]. PICK1 protein is crucial to synaptic organization of neurotransmitter systems. It acts by interacting with the C-terminal PDZ motifs of AMPA, kainate and metabotropic glutamate receptor subunits and subtypes. These complexes regulate phosphorylation of interacting partners, altering their synaptic clustering, trafficking to the neuronal surface and membrane recycling [23]. Activity dependent ubiquitination and degradation of PSD95 and other membrane-associated guanylate kinases by the E3 ligase, MDM2, have also been reported to affect the dynamics of glutamate receptor expression in postsynaptic membranes [24], [25]. It is highly likely therefore, that changes in expressional levels of PSD genes and any genes that control their turnover may affect glutamergic synaptic transmission, thereby increasing susceptibility to schizophrenia. In addition, the *DLG4* and *PICK1* genes, map to chromosome 17p13.1 and 22q13.1, respectively, regions that are known schizophrenia susceptibility loci, making them ideal positional candidates to screen for schizophrenia genetic predisposition [23], [26].

By performing a three staged genetic analysis in independent cohorts of Japanese and Chinese schizophrenia patients, our study aimed to investigate the role of genomic variants in *DLG4*, *DLG1*, *PICK1* and *MDM2* in determining the predisposition to schizophrenia.

## Materials and Methods

### Subjects

This study was performed in a three-staged manner, and included two Japanese cohorts and a cohort of Han Chinese subjects. The first stage of analysis used 124 complex pedigrees, with 80 trio samples from Japanese schizophrenia pedigrees (376 subjects) [27]. In the second stage, Chinese schizophrenia samples from 293 pedigrees (1,163 subjects: 9 trios and 284 quads) collected by the NIMH initiative (http://nimhgenetics.org/) were analyzed. In the third stage, Japanese case-control samples consisting of 4,182 unrelated individuals (2,012 schizophrenia patients, mean age ± SD = 48.13±14.40 years; 2,170 controls, mean age ± SD = 42.40±14.22 years) were studied. The case-control samples also included the probands (n = 80) from the Japanese schizophrenia trio samples. Best-estimate life-time diagnosis of patients was made by direct interview, with at least two experienced psychiatrists using the Diagnostic and Statistical Manual of Mental Disorders–IV (DSM-IV) criteria and all available information from medical records, hospital staff and family informants. Controls were recruited from hospital staff and company employees documented to be free from psychoses. Controls were interviewed by experienced psychiatrists, to exclude any past or present psychiatric disorders.

All case-control subjects were recruited from the Honshu area of Japan (the nation’s main island). Populations in Honshu fall into a single genetic cluster [28]. In our previous analysis using a subset of the same participants, *Pr* (K = 1) [namely the probability that the number of populations present in the sample = 1 [29]] was larger than 0.99 [30], [31] and λ [the genomic control factor [32]] was 1.074 [33]. These data indicated a negligible population stratification effect in our Japanese samples. All Japanese participants gave informed, written consent in a standard consent form to participate in the study after being provided with, and receiving a full explanation of study protocols and objectives. All potential participants who declined to participate or otherwise did not participate were eligible for treatment (if applicable) and were not disadvantaged in any other way by not participating in the study. The present study was approved by the Ethics Committee of RIKEN, Hamamatsu University School of Medicine and Kanazawa University Graduate School of Medicine, and conducted according to the principles expressed in the Declaration of Helsinki. DNA was extracted from whole blood according to a standard protocol for genotyping.

Post mortem brain tissues from schizophrenia and age-matched control samples were obtained from Maryland Brain Collection (http://www.mprc.umaryland.edu/mbc.asp) at the Maryland Psychiatric Research Center, Baltimore, Maryland. Frozen tissue samples from dorsolateral prefrontal cortex [Brodmann’s area 46 (BA46)] and hippocampal CA1 regions were used in this study. There were no significant demographic differences between schizophrenia and control brain samples, in terms of post mortem interval and sample pH **(Table S1 in File S1)**. The total RNA was extracted using miRNAeasy Mini kit (QIAGEN GmbH, Hilden, Germany) and the single stranded cDNA was synthesized using SuperScript VILO cDNA synthesis kit as per the manufacturer’s instructions.

### Analyzed Genes, Single Nucleotide Polymorphism (SNP) Selection and Genotyping

Four PSD protein-coding genes, *DLG4*, *DLG1*, *PICK1* and *MDM2* were selected to examine genetic predisposition to schizophrenia. The tagged SNPs for genotyping were selected to efficiently capture information on common variations in and around these genes (±10 kb). Carlson’s greedy algorithm [34] was used to make SNP list from both Chinese and Japanese populations in the HapMap database (HapMap Data Rel 20/Phase II Jan06, on NCBI B35 assembly, dbSNP b125) (http://hapmap.ncbi.nlm.nih.gov/). HapMap-Select-Processor (http://bioapp.psych.uic.edu/HapMap-LDSelect-Processor.html) was used for this SNP tagging procedure, with *r* ^2^ and the minor allele frequency threshold set to 0.85 and 0.1, respectively. A total of 32 SNPs were selected for genotyping from candidate genes **(Table S2 in File S1)**. Genotyping was performed using the TaqMan SNP Genotyping Assays (Applied Biosystems, Foster City, CA, USA) or iPlex Assay on the Sequenom MassARRAY platform (Sequenom, San Diego, CA), following the manufacturers’ instructions.

### Gene Expression Analysis

Real-time quantitative RT-PCR analysis was conducted using standard procedures, in an ABI7900HT Fast Real-Time PCR System (Applied Biosystems). TaqMan probes and primers for *DLG4* and *GAPDH* (internal control) were procured from TaqMan Gene Expression Assays (Applied Biosystems). All real-time quantitative RT-PCR reactions were performed in triplicate, based on the standard curve method.

### Statistical Analysis

The first two stages were analyzed in a family-based design and the third stage analysis was performed in an unrelated case-control design. The family-based genetic associations were tested using the Family-Based Association Test (FBAT) v2.0.3 program (http://www.biostat.harvard.edu/~fbat/) for SNPs, and *hbat* command in the same program for haplotype based association testing. The computation was performed using the Monte Carlo simulation with 100,000 replications. Correction for multiple testing on the single markers and haplotypes was performed using the false discovery rate method [35], scripted in R (http://www.r-project.org/). In the third stage analysis of the case-control replication cohort, Fisher’s exact test (two-tailed) was used to compare allele frequencies between patients and control subjects. Statistical differences in genotype distributions were evaluated using Pearson’s χ^2^ test. The linkage disequilibrium (LD) pattern was plotted for genotyped SNPs using Haploview 4.2 (http://www.broadinstitute.org/science/programs/medical-and-population-genetics/haploview/version-42-15-september-2009) and haplotype analysis was performed using Unphased 3.1.5 (http://sourceforge.net/projects/unphased/files/unphased-3.1.5.zip/download) with 10,000 permutations for deriving empirical significance. To detect significant changes in target gene expression levels among the cases and controls, t-test analysis, followed by Bonferroni correction was used. P values of <0.05 were considered significant in this study.

## Results

**Figure 1:**
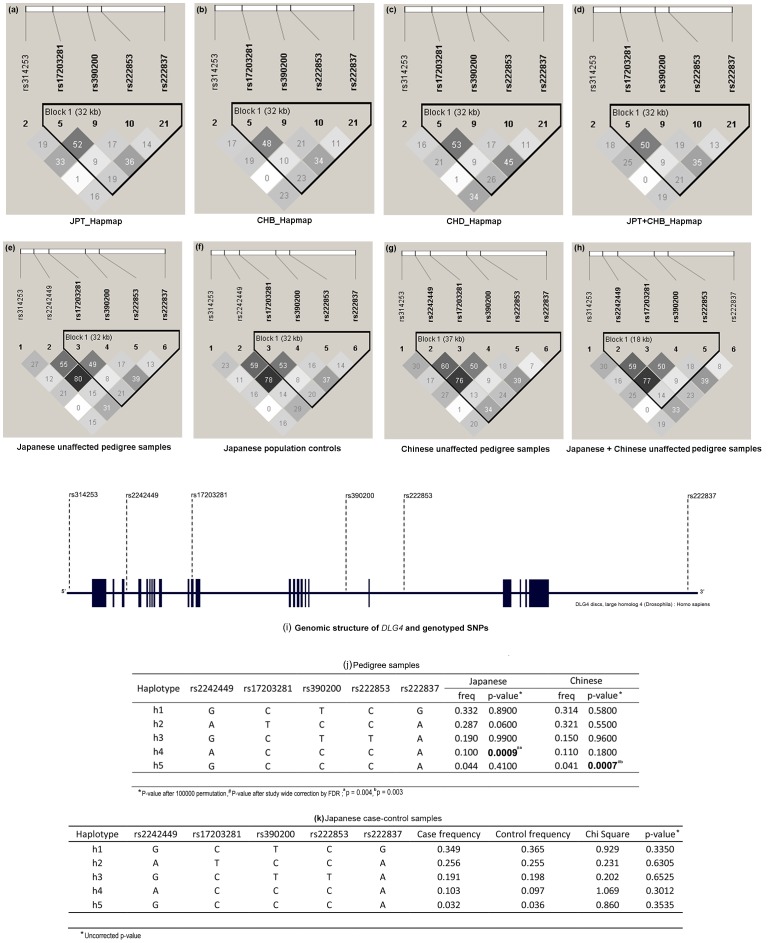
Linkage disequilibrium plots of DLG4 gene of Japanese and Chinese ancestry. (a–d) from Hapmap database, (e–h) unaffected pedigree samples and population controls of the present study,(i) Genomic structure of DLG4 and genotyped SNPs (j) DLG4 haplotypes of schizophrenia pedigree samples and (k) unrelated schizophrenia case-control sample.

To evaluate the contribution to schizophrenia susceptibility by genomic variants of genes encoding postsynaptic density proteins, 31 SNPs from four genes (*DLG4*, *DLG1*, *PICK1* and *MDM2*) were queried in schizophrenia pedigrees of Japanese descent. Owing to a genotyping failure, the SNP rs13053681, located in *PICK1* was excluded from analysis. Genotype distributions of all the studied variants were in Hardy-Weinberg equilibrium (p>0.05). FBAT analysis of Japanese schizophrenia pedigrees in the first stage, showed significant association of rs17203281 in *DLG4* with a preferential transmission of the C allele (Z = 2.239, p = 0.02) **(** **Table 1** **)**. However, this association did not survive correction for multiple testing. None of the other variants studied showed statistically significant association with disease, implying little or no role in this sample set **(Tables S3, S4 and S5 in File S1)**. Since there was a deflection of allelic transmission for rs17203281, haplotypes involving this variant was reconstructed using the EM algorithm, implemented in the FBAT package. The frequency threshold for rare haplotypes was set at 0.04. Out of the five haplotypes meeting this threshold, a five marker haplotype, h4, consisting of rs2242449-A, rs17203281-C, rs390200-C, rs222853-C and rs222837-A showed significantly excessive transmission from parents to affected offspring (Z = 3.273, p = 0.0009, after 100,000 permutation) **[** **Figure 1** **(i)].**

**Table 1:** FBAT analysis of the *DLG4* in Japanese schizophrenia pedigree samples.

Marker	Allele	Frequency	fam#	S	E(S)	Var(S)	Z	p-value	p-value (FDR)
rs314253	A	0.528	70	69	78.00	28.38	−1.68	0.09	0.27
	G	0.472	70	91	82.00	28.38	1.68	0.09	0.27
rs2242449	A	0.400	72	69	68.83	23.08	0.03	0.97	1
	G	0.600	72	93	93.16	23.08	−0.03	0.97	1
rs17203281	T	0.267	63	45	55.00	19.94	−2.23	**0.02**	0.15
	C	0.733	63	99	89.00	19.94	2.23	**0.02**	0.15
rs390200	T	0.575	69	83	82.66	22.72	0.07	0.94	1
	C	0.425	69	67	67.33	22.72	−0.07	0.94	1
rs222853	T	0.191	51	37	36.66	16.72	0.08	0.93	1
	C	0.809	51	79	79.33	16.72	−0.08	0.93	1
rs222837	A	0.628	67	82	82.00	23.50	0	1	1
	G	0.372	67	66	66.00	23.50	0	1	1

fam# = Number of nuclear families informative for the FBAT analysis.

S = Observed transmission for each allele.

E(S) = Expected transmission for each allele.

Var(S) = Variance of the observed transmission for each allele.

Z score: Positive values indicate increased transmission and negative values indicate reduced transmission to affected individuals.

FDR = P-value adjusted by False Discovery Rate.

We tested the observed allelic and haplotypic association of *DLG4* in 293 schizophrenia pedigree samples of Han Chinese ancestry. However, we could not replicate the initial association of rs17203281 in Chinese schizophrenia samples **(** **Table 2** **)**. In the Chinese population, the h4 haplotype occurs with a similar frequency to the Japanese population, but still, no statistical significant association with cases was observed. Interestingly, the less frequent haplotype, h5, comprising rs2242449-G, rs17203281-C, rs390200-C, rs222853-C and rs222837-A was found to be excessively transmitted in Chinese schizophrenia pedigrees (Z = 3.328, p = 0.0007, after 100,000 permutation).

**Table 2:** FBAT analysis of the *DLG4* in Chinese schizophrenia pedigree samples.

Marker	Allele	Frequency	fam#	S	E(S)	Var(S)	Z	p-value	p-value (FDR)
rs314253	A	0.498	214	421	406.50	137.25	1.23	0.21	0.64
	G	0.502	214	415	429.50	137.25	−1.23	0.21	0.64
rs2242449	A	0.444	215	377	396.00	138.00	−1.61	0.10	0.63
	G	0.556	215	467	448.00	138.00	1.61	0.10	0.63
rs17203281	T	0.327	195	282	291.00	123.50	−0.81	0.41	0.83
	C	0.673	195	482	473.00	123.50	0.81	0.41	0.83
rs390200	T	0.509	212	434	429.00	135.00	0.43	0.66	0.88
	C	0.491	212	394	399.00	135.00	−0.43	0.66	0.88
rs222853	T	0.157	134	162	165.00	77.50	−0.34	0.73	0.88
	C	0.843	134	364	361.00	77.50	0.34	0.73	0.88
rs222837	A	0.663	205	491	490.00	126.50	0.08	0.92	0.92
	G	0.337	205	311	312.00	126.50	−0.08	0.92	0.92

fam# = Number of nuclear families informative for the FBAT analysis.

S = Observed transmission for each allele.

E(S) = Expected transmission for each allele.

Var(S) = Variance of the observed transmission for each allele.

Z score: Positive values indicate increased transmission and negative values indicate reduced transmission to affected individuals.

FDR = P-value adjusted by False Discovery Rate.

To better understand the role of *DLG4* in schizophrenia, rs17203281 along with other variants were tested in 4,182 Japanese schizophrenia case-control samples. The results showed no significant association at allelic, genotypic **(** **Table 3** **)** or haplotype levels **[** **Figure 1** **(i)]**. The LD patterns of *DLG4* in the studied populations were comparable to the reference populations in the HapMap database **[** **Figure 1** **(a–h)].** The SNPs rs17203281, rs390200, rs222853 and rs222837 formed a LD block in Japanese population **[** **Figure 1** **(e, f)].** However the LD pattern in the Chinese population differed from that of Japanese, where the variant rs2242449 along with other SNPs formed a LD block **[** **Figure 1** **(g)].** Haplotype analysis of the other candidate genes also yielded no significant association with schizophrenia **(Tables S6, S7 and S8 in File S1)**.

**Table 3:** Case control association analysis of the *DLG4* in Japanese schizophrenia patients.

SNP	Subjects	Genotype			P-value	Allele		Odds ratio	95% Confidence Interval	P-value*
		A/A	A/G	G/G		A	G			
rs314253	Case	538(0.275)	948(0.485)	468(0.239)	0.169	2024(0.517)	1884(0.482)	1.086	0.995–1.185	0.062
	Control	626(0.294)	1041(0.489)	462(0.217)		2293(0.538)	1965(0.461)			
		A/A	A/G	G/G		A	G			
rs2242449	Case	284(0.149)	893(0.469)	725(0.381)	0.178	1461(0.384)	2343(0.615)	0.922	0.841–1.009	0.077
	Control	274(0.130)	981(0.468)	840(0.401)		1529(0.364)	2661(0.635)			
		C/C	C/T	T/T		C	T			
rs17203281	Case	1064(0.544)	740(0.378)	149(0.0762)	0.784	2868(0.734)	1038(0.265)	1.020	0.924–1.126	0.693
	Control	1176(0.553)	782(0.368)	165(0.0777)		3134(0.738)	1112(0.261)			
		C/C	C/T	T/T		C	T			
rs390200	Case	347(0.178)	910(0.467)	691(0.354)	0.471	1604(0.411)	2292(0.588)	0.956	0.874–1.044	0.315
	Control	348(0.163)	1008(0.474)	770(0.362)		1704(0.400)	2548(0.599)			
		C/C	C/T	T/T		C	T			
rs222853	Case	1249(0.641)	592(0.304)	105(0.053)	0.470	3090(0.793)	802(0.206)	0.998	0.896–1.111	0.966
	Control	1348(0.634)	675(0.317)	101(0.047)		3371(0.793)	877(0.206)			
		A/A	A/G	G/G		A	G			
rs222837	Case	735(0.385)	903(0.473)	270(0.141)	0.993	2373(0.621)	1443(0.378)	0.995	0.908–1.089	0.911
	Control	803(0.383)	992(0.474)	298(0.142)		2598(0.620)	1588(0.379)			

* Uncorrected p-value.

**Figure 2:**
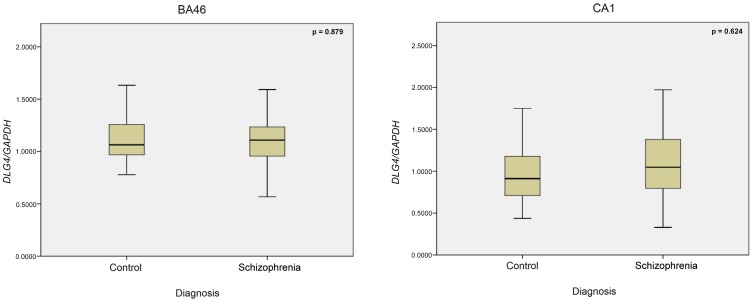
DLG4 gene expression analysis in BA46 and CA1 region of postmortem brain samples from schizophrenia patients and controls.

Since an allelic association was observed for *DLG4*, gene expression analysis in the dorsolateral prefrontal cortex, BA46 and hippocampal CA1 regions of schizophrenia patient samples was performed, but showed no significant changes between patient and control samples **(** **Figure 2** **).**

## Discussion

The PSD of glutamatergic synapses harbors numerous proteins, which maintain synaptic dynamics and thereby, synaptic plasticity. The PSD95 group (DLG 1–4) constitutes the major component in the postsynaptic density of glutamatergic synapses. This group differentially regulates basal synaptic activity by affecting connections between receptors and their effectors.

In this study, we first performed family-based association analysis of genetic variants in the PSD genes; *DLG4*, *DLG1*, *PICK1* and *MDM2* using Japanese schizophrenia pedigrees. Although initial analysis showed significant association of the synonymous *DLG4* variant, rs17203281, with over-transmission of the C allele, this association result could not be replicated in an ethnically close Chinese pedigree sample set, or in a Japanese schizophrenia case-control cohort. These results are in line with previous observations in Japanese and Chinese populations, which found no association of *DLG4* with disease [36], [37]. Moreover, there was no aberrant change in *DLG4* gene expression in schizophrenia brain tissues.

However, we did find significant association of the h4 haplotype, consisting of rs2242449-A, rs17203281-C, rs390200-C, rs222853-C and rs222837-A, observed in Japanese, but not Chinese schizophrenia pedigree samples. So, even though both populations show a similar haplotype frequency, there is specific enrichment of haplotypes in Japanese cases. Interestingly, the h5 haplotype, which differs from the Japanese risk haplotype at the first allele, was significantly associated with schizophrenia in the Chinese population.

These results showed that the combination of rs17203281-C, rs390200-C, rs222853-C and rs222837-A, formed the core haplotype in both populations. However, the variant rs2242449 was deemed to determine over-transmission of the haplotypes to affected offspring in schizophrenia family samples. In the Japanese cohort, the 5 window haplotype containing rs2242449-A along with the core haplotype was found to be risk-conferring **(** **Table 4** **)**. In the Chinese cohort, the rs2242449-G allele, along with the core haplotype showed significant over-transmission. This observation further underscores the possibility of a population specific lineage of haplotypes determining disease risk. Further, explorations of ENCODE database annotations (HaploReg v2, http://www.broadinstitute.org/mammals/haploreg/haploreg.php and RegulomeDB, http://regulome.stanford.edu/index) for regulatory effects, revealed that the variant rs2242449, would affect the regulatory motifs, GATA and TATA, as well as binding of ZNF263 protein in T-REx-HEK293 cell lines **(Figure S1).** At this point, the effects in neuronal cells are unknown and our association results suggest no substantial regulatory effect for rs2242449 in brain cells, at least in terms of schizophrenia manifestation.

**Table 4:** *DLG4* haplotype comparison in Japanese and Chinese schizophrenia pedigree samples.

Ethnicity	Haplotype	rs314253	rs2242449	rs17203281	rs390200	rs222853	rs222837	Allele frequency	P-value	Z
Japanese	a1	G	A	C	C	C	A	0.095	**0.0082**	2.697
	a2		**A**	C	C	C	A	0.104	**0.0008**	3.273
	a3			C	C	C	A	0.151	**0.0032**	3.051
Chinese	a1	G	A	C	C	C	A	0.089	0.1846	−1.324
	a2		**G**	C	C	C	A	0.041	**0.0007**	3.328
	a3			C	C	C	A	0.151	0.5276	0.638

This study suggests that rs17203281 may be in linkage disequilibrium with a causal variant or variants, located in the regulatory elements of *DLG4* and that the haplotype, including rs17203281 may span the putative causal variant(s). The role of the *DLG4* haplotype in schizophrenia susceptibility has been further substantiated in a recent study using Taiwanese cohorts, which showed association of a haplotype spanning the core promoter and 5′-UTR regions with disease [15]. These findings all advocate dense screening for variants and haplotypes to clarify the role of *DLG4* in schizophrenia.

Even though expression level changes of *DLG4* have been reported in schizophrenia brains [15], these findings conflict with previous study results. In this study, we did not observe any significant changes in *DLG4* expression levels within the brain regions of BA46 and CA1 in schizophrenia patients, a result which mirrors the observations of a recent study [38]. There is a possibility that *DLG4* gene expression could be modulated by polypyrimidine tract binding (PTB) proteins [39]. In fact, two genome wide association studies have reported the association of *PTBP2* in European schizophrenia patients [40], [41].

Although numerous studies have shown expression level changes and genetic association between other PSD genes and schizophrenia [42]–[48], our study failed to detect association between *DLG1*, *PICK1* and *MDM2,* and schizophrenia in Japanese cohorts, which is in line with reports for *DLG1* [49] and *PICK1* [50].

One of the limitations of our study is the limited number of SNPs queried from candidate genes, based on tagging status. This raises the potential for missing rare variants with substantial effect sizes. Future studies should focus on dense screening in selected haplotype blocks. Moreover haplotypes tend to be conserved through evolutionary processes and can also capture potential *cis*-interacting variants [51]. Another limitation would be the relatively small sample size of the pedigrees, coupled with an inherent power deficit in family based study designs. The non-replication of the association seen in large sized case control cohorts may suggest only modest effect of the haplotype.

To conclude, our study identified haplotypes in *DLG4* that confer a risk for schizophrenia in Japanese and Chinese populations. Future studies should focus on narrowing down further the region in and around this haplotype for potential disease causative variants.

## Supporting Information

Figure S1 ENCODE database annotations for rs2242449, affecting the regulatory motifs.
(TIF)

File S1 Table S1: Clinical characteristics of Post mortem tissue samples from BA46 and CA1 region obtained from Maryland Brain Collection (http://www.mprc.umaryland.edu/mbc.asp) at the Maryland Psychiatric Research Center, Baltimore, Maryland. Table S2: SNP genotyped in the study subjects. Table S3: FBAT analysis of the DLG1 in Japanese pedigrees. Table S4: FBAT analysis of the PICK1 in Japanese pedigrees. Table S5: FBAT analysis of the MDM2 in Japanese pedigrees. Table S6: Haplotype analysis of the DLG1 in Japanese pedigrees. Table S7: Haplotype analysis of the PICK1 in Japanese pedigrees. Table S8: Haplotype analysis of the MDM2 in Japanese pedigrees.
(DOCX)
