# Supplementary figures and images for: Population-Specific Haplotype Association of the Postsynaptic Density Gene DLG4 with Schizophrenia, in Family-Based Association Studies
Source: PLoS One. 2013 Jul 25;8(7):e70302. doi: 10.1371/journal.pone.0070302 (PMC3723755; doi:10.1371/journal.pone.0070302)

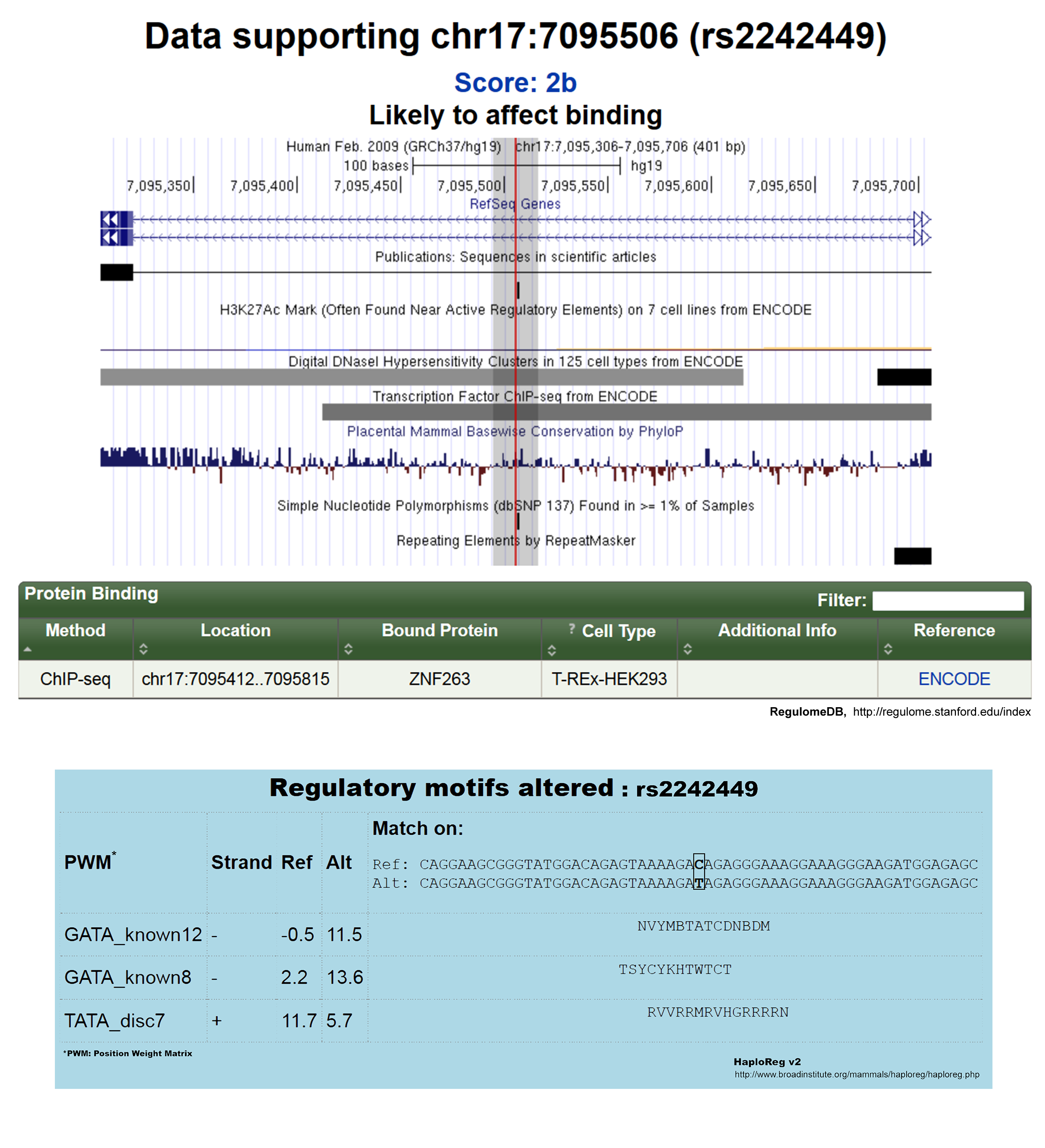

Supplement: Figure S1 — ENCODE database annotations for rs2242449, affecting the regulatory motifs. (TIF) [file pone.0070302.s001.tif]
