# Supplementary material for: Population-Specific Haplotype Association of the Postsynaptic Density Gene DLG4 with Schizophrenia, in Family-Based Association Studies
Source: PLoS One. 2013 Jul 25;8(7):e70302. doi: 10.1371/journal.pone.0070302 (PMC3723755; doi:10.1371/journal.pone.0070302)
Supplement: File S1 — Table S1: Clinical characteristics of Post mortem tissue samples from BA46 and CA1 region obtained from Maryland Brain Collection (http://www.mprc.umaryland.edu/mbc.asp) at the Maryland Psychiatric Research Center, Baltimore, Maryland. Table S2: SNP genotyped in the study subjects. Table S3: FBAT analysis of the DLG1 in Japanese pedigrees. Table S4: FBAT analysis of the PICK1 in Japanese pedigrees. Table S5: FBAT analysis of the MDM2 in Japanese pedigrees. Table S6: Haplotype analysis of the DLG1 in Japanese pedigrees. Table S7: Haplotype analysis of the PICK1 in Japanese pedigrees. Table S8: Haplotype analysis of the MDM2 in Japanese pedigrees. (DOCX) [file pone.0070302.s002.docx]

| **Brain Region** | **Diagnosis** | **Storage time (years)*** | **Age*** | **pH*** | **PMI*** | **Gender, n (%)** | | **Ethnicity, n (%)** | |
| --- | --- | --- | --- | --- | --- | --- | --- | --- | --- |
|  |  |  |  |  |  | **Female** | **Male** | **Black** | **White** |
| BA46 | Control (n= 35) | 16.60 ± 4.49 | 45.85 ± 13.03 | 6.60 ± 0.20 | 13.65 ± 6.36 | 13(0.371) | 22(0.628) | 14(0.400) | 21(0.600) |
| BA46 | Schizophrenia (n= 35) | 15.68 ± 5.40 | 45.51 ± 13.59 | 6.56 ± 0.44 | 14.20 ± 6.57 | 9(0.257) | 26(0.743) | 11(0.314) | 24(0.686) |
| CA1 | Control (n=20) | 15.00 ± 4.01 | 42.40 ± 12.30 | 6.60 ± 0.24 | 15.10 ± 6.71 | 7(0.350) | 13(0.650) | 9(0.450) | 11(0.550) |
| CA1 | Schizophrenia (n=20) | 12.85 ± 5.42 | 45.00 ± 15.34 | 6.37 ± 0.37 | 14.70 ± 5.92 | 5(0.250) | 15(0.750) | 5(0.250) | 15(0.750) |
|  |  |  |  |  |  |  |  |  |  |

PMI=Post mortem interval, * Mean ± SD

Table S1: Clinical characteristics of Post mortem tissue samples from BA46 and CA1 region obtained from Maryland Brain Collection (http://www.mprc.umaryland.edu/mbc.asp) at the Maryland Psychiatric Research Center, Baltimore, Maryland

| **Gene Name** | **SNP** | **Position*** | **Alleles** | **Assay ID** |
| --- | --- | --- | --- | --- |
| *DLG4* chr17:7033936-7063745 Genomic Size: 29,810 (-) | rs314253 | 7,032,374 | A/G | C___1505686_10 |
|  | rs2242449 | 7,036,231 | A/G | C___1505681_1_ |
|  | rs17203281 | 7,040,535 | C/T | C__22272552_10 |
|  | rs390200 | 7,050,719 | T/C | C___2552959_10 |
|  | rs222853 | 7,054,704 | C/T | C___2261180_10 |
|  | rs222837 | 7,073,280 | G/A | C___2261188_10 |
| *DLG1* chr3:198259733-198513757 Genomic Size: 254,025 (-) | rs7623862 | 198,256,490 | T/C | C__29879763_10 |
|  | rs7636654 | 198,257,066 | C/G |  |
|  | rs7638423 | 198,262,666 | C/T | C___3061892_10 |
|  | rs1949471 | 198,353,552 | G/A | C___8812811_10 |
|  | rs12152266 | 198,399,671 | G/A | C___1333279_10 |
|  | rs2122824 | 198,442,757 | A/C | C___1333364_10 |
|  | rs9843659 | 198,473,906 | G/A | C__29699165_10 |
|  | rs338222 | 198,520,534 | C/G | C____619121_10 |
|  | rs338223 | 198,521,918 | A/T |  |
|  | rs6764023 | 198,523,295 | T/G |  |
| *PICK1* chr22:36777903-36796207 Genomic Size: 18,305 (+) | rs5756890 | 36,769,900 | A/C |  |
|  | rs4821733 | 36,773,894 | A/G | C___2487449_10 |
|  | rs8142185 | 36,775,580 | A/C | C___2487454_1_ |
|  | rs13053681 | 36,784,785 | C/G | Failed to design |
|  | rs3026688 | 36,787,641 | A/C | C___2487473_10 |
|  | rs2076369 | 36,788,152 | G/T | C___2487476_1_ |
|  | rs760975 | 36,792,284 | C/G | C_____12903_20 |
|  | rs2012859 | 36,798,206 | A/G | C___2487482_1_ |
|  | rs2076371 | 36,799,196 | A/G | C__16167960_10 |
|  | rs2076372 | 36,799,465 | C/T | C___2487485_10 |
|  | rs2235573 | 36,802,430 | A/G | C__22272536_10 |
|  | rs2267366 | 36,805,159 | C/T | C___2487491_10 |
| *MDM2* chr12:67488247-67520481 Genomic Size: 32,235 (+) | rs10748111 | 67,479,823 | C/T | C__32171809_10 |
|  | rs1470383 | 67,493,429 | G/A | C___7469105_10 |
|  | rs2291857 | 67,504,305 | T/G | C__25474795_10 |
|  | rs1690916 | 67,521,673 | A/G |  |
|  |  |  |  |  |

*UCSC (http://genome.ucsc.edu/), Human Feb. 2009 (GRCh37/hg19) Assembly

Table S2: SNP genotyped in the study subjects

|  | | |  |  |  |  |  |
| --- | --- | --- | --- | --- | --- | --- | --- |
| Marker | Allele | Frequency | fam# | S-E(S) | Var(S) | Z | p-value |
| rs6764023 | T | 0.833 | 42 | -3.33 | 12.72 | -0.93 | 0.35 |
|  | G | 0.167 | 42 | 3.33 | 12.72 | 0.93 | 0.35 |
| rs338223 | A | 0.827 | 42 | 3.66 | 14.55 | 0.96 | 0.33 |
|  | T | 0.173 | 42 | -3.66 | 14.55 | -0.96 | 0.33 |
| rs338222 | G | 0.351 | 64 | -3.00 | 23.38 | -0.62 | 0.53 |
|  | C | 0.649 | 64 | 3.00 | 23.38 | 0.62 | 0.53 |
| rs9843659 | A | 0.629 | 71 | 3.00 | 25.38 | 0.59 | 0.55 |
|  | G | 0.371 | 71 | -3.00 | 25.38 | -0.59 | 0.55 |
| rs2122824 | A | 0.22 | 48 | 1.83 | 14.97 | 0.47 | 0.63 |
|  | C | 0.78 | 48 | -1.83 | 14.97 | -0.47 | 0.63 |
| rs12152266 | A | 0.552 | 70 | 2.00 | 24.88 | 0.40 | 0.68 |
|  | G | 0.448 | 70 | -2.00 | 24.88 | -0.40 | 0.68 |
| rs1949471 | A | 0.111 | 37 | -4.16 | 12.47 | -1.18 | 0.23 |
|  | G | 0.889 | 37 | 4.16 | 12.47 | 1.18 | 0.23 |
| rs7638423 | T | 0.659 | 65 | 2.00 | 24.00 | 0.40 | 0.68 |
|  | C | 0.341 | 65 | -2.00 | 24.00 | -0.40 | 0.68 |
| rs7636654 | G | 0.124 | 40 | -5.16 | 13.30 | -1.41 | 0.15 |
|  | C | 0.876 | 40 | 5.16 | 13.30 | 1.41 | 0.15 |
| rs7623862 | T | 0.171 | 45 | 1.83 | 12.97 | 0.50 | 0.61 |
|  | C | 0.829 | 45 | -1.83 | 12.97 | -0.50 | 0.61 |
|  |  |  |  |  |  |  |  |

fam# = Number of nuclear families informative for the FBAT analysis.

S-E(S) = Observed minus the expected transmission for each allele.

Var(S) = Variance of the observed transmission for each allele.

Z score: Positive values indicate increased transmission and negative values indicate reduced transmission to affected individuals.

Table S3: FBAT analysis of the *DLG1* in Japanese pedigrees

|  | | |  |  |  |  |  |
| --- | --- | --- | --- | --- | --- | --- | --- |
| Marker | Allele | Frequency | fam# | S-E(S) | Var(S) | Z | p-value |
| rs5756890 | A | 0.167 | 61 | -4.50 | 20.25 | -1 | 0.31 |
|  | C | 0.833 | 61 | 4.50 | 20.25 | 1 | 0.31 |
| rs4821733 | A | 0.493 | 67 | 1.33 | 22.72 | 0.28 | 0.78 |
|  | G | 0.507 | 67 | -1.33 | 22.72 | -0.28 | 0.78 |
| rs8142185 | A | 0.671 | 58 | 1.00 | 19.88 | 0.22 | 0.82 |
|  | C | 0.329 | 58 | -1.00 | 19.88 | -0.22 | 0.82 |
| rs3026688 | A | 0.150 | 46 | 2.83 | 14.36 | 0.74 | 0.45 |
|  | C | 0.850 | 46 | -2.83 | 14.36 | -0.74 | 0.45 |
| rs2076369 | T | 0.461 | 70 | -1.83 | 22.97 | -0.38 | 0.70 |
|  | G | 0.539 | 70 | 1.83 | 22.97 | 0.38 | 0.70 |
| rs760975 | G | 0.357 | 60 | 1.00 | 21.88 | 0.21 | 0.83 |
|  | C | 0.643 | 60 | -1.00 | 21.88 | -0.21 | 0.83 |
| rs2012859 | A | 0.204 | 55 | 2.33 | 17.11 | 0.56 | 0.57 |
|  | G | 0.796 | 55 | -2.33 | 17.11 | -0.56 | 0.57 |
| rs2076371 | A | 0.181 | 41 | -0.33 | 12.22 | -0.09 | 0.92 |
|  | G | 0.819 | 41 | 0.33 | 12.22 | 0.09 | 0.92 |
| rs2076372 | T | 0.327 | 56 | 3.50 | 19.63 | 0.79 | 0.43 |
|  | C | 0.673 | 56 | -3.50 | 19.63 | -0.79 | 0.43 |
| rs2235573 | A | 0.471 | 70 | 5.00 | 24.50 | 1.01 | 0.31 |
|  | G | 0.529 | 70 | -5.00 | 24.50 | -1.01 | 0.31 |
| rs2267366 | T | 0.714 | 57 | 3.00 | 20.00 | 0.67 | 0.50 |
|  | C | 0.286 | 57 | -3.00 | 20.00 | -0.67 | 0.50 |
|  |  |  |  |  |  |  |  |

fam# = Number of nuclear families informative for the FBAT analysis.

S-E(S) = Observed minus the expected transmission for each allele.

Var(S) = Variance of the observed transmission for each allele.

Z score: Positive values indicate increased transmission and negative values indicate reduced transmission to affected individuals.

Table S4: FBAT analysis of the *PICK1* in Japanese pedigrees

| Marker | Allele | Frequency | fam# | S-E(S) | Var(S) | Z | P |
| --- | --- | --- | --- | --- | --- | --- | --- |
| rs10748111 | T | 0.575 | 66 | 2.50 | 23.25 | 0.51 | 0.60 |
|  | C | 0.425 | 66 | -2.50 | 23.25 | -0.51 | 0.60 |
| rs1470383 | A | 0.856 | 42 | -0.83 | 13.47 | -0.22 | 0.82 |
|  | G | 0.144 | 42 | 0.83 | 13.47 | 0.22 | 0.82 |
| rs2291857 | T | 0.294 | 65 | -3.50 | 22.69 | -0.73 | 0.46 |
|  | G | 0.706 | 65 | 3.50 | 22.69 | 0.73 | 0.46 |
| rs1690916 | A | 0.267 | 55 | -3.00 | 19.50 | -0.67 | 0.49 |
|  | G | 0.733 | 55 | 3.00 | 19.50 | 0.67 | 0.49 |
|  |  |  |  |  |  |  |  |

fam# = Number of nuclear families informative for the FBAT analysis.

S-E(S) = Observed minus the expected transmission for each allele.

Var(S) = Variance of the observed transmission for each allele.

Z score: Positive values indicate increased transmission and negative values indicate reduced transmission to affected individuals.

Table S5: FBAT analysis of the *MDM2* in Japanese pedigrees

| Haplotype | rs6764023 | rs338223 | rs338222 | rs9843659 | rs2122824 | rs12152266 | rs1949471 | rs7638423 | rs7636654 | rs7623862 | Frequency | Z | p-value |
| --- | --- | --- | --- | --- | --- | --- | --- | --- | --- | --- | --- | --- | --- |
| a1 | T | A | C | A | C | A | G | T | C | C | 0.479 | 1.254 | 0.200 |
| a2 | T | T | G | G | C | G | A | C | G | C | 0.108 | -0.886 | 0.375 |
| a3 | G | A | G | G | A | G | G | C | C | T | 0.091 | 1.844 | 0.065 |
| a4 | T | A | C | A | C | G | G | T | C | C | 0.051 | 0.810 | 0.418 |
| a5 | T | T | G | G | A | G | G | C | C | T | 0.043 | -0.253 | 0.800 |
|  |  |  |  |  |  |  |  |  |  |  |  |  |  |

Z score: Positive values indicate increased transmission and negative values indicate reduced transmission to affected individuals.

Table S6: Haplotype analysis of the *DLG1* in Japanese pedigrees

| Haplotype | rs5756890 | rs4821733 | rs8142185 | rs3026688 | rs2076369 | rs760975 | rs2012859 | rs2076371 | rs2076372 | rs2235573 | rs2267366 | Frequency | Z | p-value |
| --- | --- | --- | --- | --- | --- | --- | --- | --- | --- | --- | --- | --- | --- | --- |
| a1 | A | A | A | A | G | C | A | G | T | A | T | 0.133 | 1.236 | 0.216 |
| a2 | A | G | A | C | T | G | G | G | T | A | T | 0.124 | -1.045 | 0.296 |
| a3 | A | A | C | C | G | C | G | G | C | A | T | 0.121 | 0.588 | 0.556 |
| a4 | A | A | C | C | G | C | G | G | C | G | T | 0.116 | -0.161 | 0.872 |
| a5 | C | G | A | C | T | G | G | A | C | G | C | 0.104 | 0.557 | 0.577 |
| a6 | A | A | C | C | G | C | G | G | C | G | C | 0.067 | -0.602 | 0.547 |
| a7 | C | G | A | C | T | G | G | A | C | G | T | 0.051 | -1.270 | 0.204 |
| a8 | C | G | A | C | T | C | G | G | C | G | C | 0.045 | 1.022 | 0.307 |
| a9 | C | G | A | C | T | G | G | G | T | A | T | 0.042 | 2.324 | 0.020 |
| a10 | C | G | A | C | G | C | A | G | C | G | T | 0.040 | 0 | 1 |
|  |  |  |  |  |  |  |  |  |  |  |  |  |  |  |

Z score: Positive values indicate increased transmission and negative values indicate reduced transmission to affected individuals.

Table S7: Haplotype analysis of the *PICK1* in Japanese pedigrees

| Haplotype | rs10748111 | rs1470383 | rs2291857 | rs1690916 | Frequency | Z | p-value |
| --- | --- | --- | --- | --- | --- | --- | --- |
| a1 | T | A | G | G | 0.583 | 0.041 | 0.96 |
| a2 | C | A | T | A | 0.166 | -1.048 | 0.29 |
| a3 | C | G | T | G | 0.105 | 0.462 | 0.64 |
| a4 | C | A | G | A | 0.088 | 0.695 | 0.48 |
|  |  |  |  |  |  |  |  |

Z score: Positive values indicate increased transmission and negative values indicate reduced transmission to affected individuals.

Table S8: Haplotype analysis of the *MDM2* in Japanese pedigrees
